# The Effect of Fear of the COVID-19 on Depression Among Chinese Outbound Students Studying Online in China Amid the COVID-19 Pandemic Period: The Role of Resilience and Social Support

**DOI:** 10.3389/fpsyg.2021.750011

**Published:** 2021-10-15

**Authors:** Yikang Chen, Yifan Liu, Yuxuan Zhang, Zheng Li, Tianshu Zhou

**Affiliations:** ^1^Department of Social and Behavioural Sciences, City University of Hong Kong, Kowloon, Hong Kong, SAR China; ^2^Department of Asian and International Studies, City University of Hong Kong, Kowloon, Hong Kong, SAR China

**Keywords:** fear of COVID-19, depression, resilience, social support, Chinese outbound students, COVID-19 pandemic

## Abstract

**Objective:** The present study focused on examining fear of the coronavirus disease 2019 (COVID-19) is correlated with depression and explored the potential role of resilience and social support on the association between fear of the COVID-19 (FoC) and depression among Chinese outbound students studying online in China amid the COVID-19 pandemic period.

**Methods:** A total of 476 Chinese outbound students from different universities worldwide, currently studying *via* online mode in China, completed an online survey including measures on FoC, resilience, social support, and depression.

**Results:** (1) Fear of the COVID-19 was positively correlated with depression and negatively correlated with resilience and social support. Both resilience and social support were negatively correlated with depression. Social support showed a positive correlation with resilience. (2) The effect of FoC on depression mainly occurred through two paths: the mediating effect of resilience and the moderating effect of resilience. However, the moderating effect of social support on the association between FoC and depression was not sustained in this study.

**Conclusion:** This study indicated the mediating and moderating effects of resilience on the association between FoC and depression among Chinese outbound students studying online in China during the COVID-19 pandemic period. The current findings confirmed that resilience has significant implications in preventing negative mental states under the COVID-19 context among this particular group.

## Introduction

Due to the rapid spread of the coronavirus disease 2019 (COVID-19) pandemic, governments worldwide have imposed travel restrictions and most higher education institutions have switched to online learning (Sahu, 2020; Walke et al., 2020). However, these rapid changes have caused a drastic increase in psychological problems for college students worldwide and put international students into a problematic situation (Al-Maroof et al., 2020; Pierce et al., 2020; Wang et al., 2020).

Moreover, the stressful situation under the COVID-19 pandemic period has severely harmed the mental health of the Chinese students studying outbound (Ma and Miller, 2020). In addition to the COVID-19 pandemic-related concerns, the Chinese outbound students (COSs) also confront isolation and discrimination as a result of China being the first country to experience the COVID-19 pandemic period (Zhai and Du, 2020), which has disrupted their studies and prompted a slew of psychological disorders such as fear and depression. Due to the unpredictability of the COVID-19 pandemic, many COSs have returned to mainland China to learn *via* online mode (Mok et al., 2021). Therefore, those COSs studying online in China have been a brand new and unique group and the study on their psychological status influenced by the COVID-19 pandemic is urgently important.

Fear of the COVID-19 (FoC) is a negative emotion or a negative response toward any danger or health threats such as harm to oneself physically, any discrimination, or isolation related to the COVID-19 (Witte and Allen, 2000; Zhang et al., 2020). Depression is a clinical mental illness comprised of a negative emotional state and is the most mainstream consequence of the COVID-19 pandemic (Kendall et al., 1987; Rajkumar, 2020). Many individuals are susceptible to depression as a result of the COVID-19 pandemic and subsequent events (Tang et al., 2020). Therefore, we speculated that FoC may be associated with depression. The present study focused on examining whether FoC is correlated with depression among COSs studying online amid the COVID-19 pandemic period. Moreover, previous FoC-related research has found that FoC may indirectly affect mental health issues *via* other resources (Rodríguez-Hidalgo et al., 2020; Belen, 2021). However, few studies extended to the effects of the resilience and social support of an individual on depression during the COVID-19 pandemic period. Aneshensel and Stone (1982) point out that social support has a practical buffering effect in preventing depressive symptoms. Meanwhile, resilience can also operate as an inner resource against negative psychological symptoms when an individual is faced with adversity. Thus, based on the buffering model of social support (Aneshensel and Stone, 1982) and resilience theory (Van Breda, 2001), this study also explored the potential role of resilience and social support on the association between FoC and depression among the COSs studying online amid the COVID-19 pandemic period.

### Fear of the COVID-19 and Depression

Previous research has shown that there is a significant positive correlation between fear of FoC and depression (Bendau et al., 2021; Sakib et al., 2021). Kaparounaki et al. (2020) found a high increase in overall depression score with increased suicidal thoughts and other anxiety symptoms throughout the first period of nationwide lockdown in Greece. Increasing FoC in the COSs caused by the quarantine and lockdown restrictions has resulted in rising uncertainties among students with regard to their academic and career efforts (Feng et al., 2021; Yang et al., 2021), and misinformation during the COVID-19 pandemic was also found to be related to FoC (Gabarron et al., 2021). Previous research has shown that FoC triggers depression (Egunjobi, 2020; Al Majali and Alghazo, 2021; Pak et al., 2021). A growing body of research provides evidence that the COVID-19-related fear is a threat to mental health and higher FoC is linked with an increased depression (Ahorsu et al., 2020; Bakioğlu et al., 2020; Yıldırım et al., 2021). Saricali et al. (2020) point out that FoC puts individuals in a high-tension state and makes them feel helpless leading to depression. Therefore, this study hypothesized that:

Hypothesis 1 (H1): FoC will be positively correlated with depression.

### The Role of Resilience

The ability of a person to recover from the traumatic events is referred to as resilience, also called the capacity to learn to live in times of fear and uncertainty and the ability to adjust to the difficult and problematic experiences in life (Meichenbaum, 2005; Herrman et al., 2011). Past studies have shown that resilience has the ability to reduce negative emotions such as depression (Songprakun and McCann, 2015; Anyan and Hjemdal, 2016). While psychological consequences still differ among people, resilience plays a crucial role in describing such individual differences. It can be termed either as an attribute, a result, or a process (Fletcher and Sarkar, 2013). Luthar et al. (2000) describe resilience as a changing approach that encompasses adjustment in times of adverse circumstances. Individuals with higher resilience can better recover from the trauma experiences (Schaubroeck et al., 2011).

**FIGURE 1:**
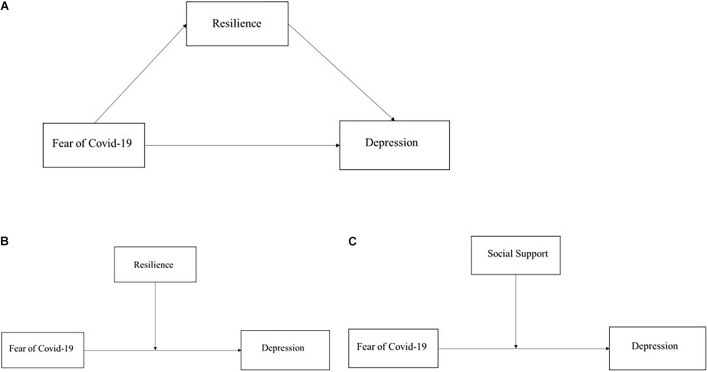
Conceptual models. **(A)** Conceptual model for testing whether resilience will mediate the association between FoC and depression. **(B)** Conceptual model for testing whether resilience will moderate the association between FoC and depression. **(C)** Conceptual model for testing whether social support will moderate the association between FoC and depression.

However, the role of resilience in the association between FoC and depression is not clear. Previous research has shown that resilience plays a mediating role in the relationship between FoC and negative mental states such as stress (Peker and Cengiz, 2021), anxiety (Seçer et al., 2020), and distress (Lorente et al., 2021). Resilience represents the certain traits that allow a person to adjust to the events that they experience (Connor and Davidson, 2003), and it has a mediating effect between fear and depression (Seçer et al., 2020), especially during the COVID-19 pandemic period (Yıldırım et al., 2020). Therefore, the present study proposed that those COSs with lower levels of resilience would have higher levels of depression. Thus, this study aimed to explore that:

Hypothesis 2 (H2): Whether low levels of resilience will mediate the association between FoC and depression [as shown in the conceptual framework in Figure 1A].

On the other hand, the existing studies have also revealed the moderating effect of resilience on depression (Wingo et al., 2010; Catalano et al., 2011). Havnen et al. (2020) demonstrated that resilience worked as a moderator in relieving depressive symptoms throughout the COVID-19 pandemic period. Meanwhile, recent empirical studies indicated that when resilience works as a moderator, it has a buffering effect on depression caused by the negative consequences associated with the COVID-19 pandemic (Aguiar-Quintana et al., 2021; Schierberl Scherr et al., 2021). Considering the buffering effect of resilience on depression, the present research also proposed to explore the potential buffering effects of resilience on the relationship between the FoC and depression of the COSs studying online in China. Thus, this study aimed to explore that:

Hypothesis 3 (H3): Whether the low levels of resilience will moderate the association between FoC and depression (as shown in the conceptual framework in Figure 1B).

### The Moderating Role of Social Support

Social support has been shown to function as a buffering variable in reducing the negative consequences of mental illness (Cohen and Wills, 1985; Trepte et al., 2015). Hornstein and Eisenberger’s (2017) research (2017) illustrates that social support has a preventative effect on the fear-related adverse effects; within this context, social support is essential to help aid the psychological problems (Li et al., 2021). College students with lower levels of social support are more likely to suffer from mental illnesses (Hefner and Eisenberg, 2009). Previous research has shown that social support is negatively correlated with depression (McDougall et al., 2016).

Besser and Priel (2008) found that social support affects depression by acting as a moderator of death-related fears, but not the COVID-19-related fears. However, a previous study revealed that social support is an influencing moderator of depression in the Chinese student groups (Zhou et al., 2013; Hou et al., 2020). Therefore, the present study argues that for the COSs with less social support, their depression levels are higher than those the COSs with lower FoC. Although previous research has shown that social support has a buffering effect on depression (Rueger et al., 2016), less support is extended to the influence of FoC. Thus, based on the previous evidence, this study hypothesized that:

Hypothesis 4 (H4): Low levels of social support will moderate the association between FoC and depression (as shown in the conceptual framework in Figure 1C).

## Materials and Methods

### Participants and Procedure

Considering the social-distancing requirement during the COVID-19 pandemic period, all the data were collected through an online self-administered survey questionnaire. This study involved 476 COSs, including 164 males (34.5%) and 312 females (65.5%), currently studying *via* online mode in China from the different universities worldwide. The survey was conducted from March 2021 to May 2021 and all the participants had returned to China before January 2021. For the age range, 243 participants ranged from 18 to 22 years (51.1%), 206 participants ranged from 23 to 27 years (43.3%), and 27 participants were older than 27 years (5.7%). Meanwhile, 238 participants were undergraduate students (50.0%) and 238 participants were graduate students, which including 213 participants at the master level (44.7%) and 25 participants at the doctoral level (5.3%). The reporting studying area indicates that 31.1% (*n* = 148) of participants were studying in Hong Kong special administrative region (SAR)/Macau SAR/Taiwan of China, 27.7% (*n* = 132) of participants were studying in North America (including the United States and Canada), 19.1% (*n* = 91) of participants were studying in the United Kingdom or European Union countries, 16.6% (*n* = 79) of participants were studying in the Asian countries (e.g., Japan, South Korea), 5.3% (*n* = 25) of participants were studying in Australia or New Zealand, and 0.2% (*n* = 1) of participants were studying in the other regions. The demographic information is shown in Table 1.

**TABLE 1:** Demographic information of the participants (*N* = 476).

Variable	*N*	Percent
**Sex**		
Males	164	34.5%
Females	312	65.5%
**Age Range**		
18 to 22 years	243	51.1%
23 to 27 years	206	43.3%
above 27 years	27	5.7%
**Education Level**		
Undergraduate	238	50.0%
Master Level	213	44.7%
Doctoral Level	25	5.3%
**Studying Region**		
HK/MO/TW	148	27.7%
North America	132	27.7%
UK/EU	91	19.1%
Asia	79	16.6%
Australia/New Zealand	25	5.3%
Others	1	0.2%

*North America including The United States of America and Canada; HK, Hong Kong SAR, China; MO, Macao SAR, China; TW, Taiwan, China; UK, The United Kingdom of Great Britain and Northern Ireland; EU, Countries in the European Union; Asia, Asian countries (excluding China).*

## Measures

### Demographic Information

Participants provided age (1 = ranged from 18 to 22 years; 2 = ranged from 23 to 27 years; 3 = older than 27 years), sex (0 = male; 1 = female), education level (1 = undergraduate students; 2 = master-level students; 3 = doctoral-level students), and outbound studying region.

### Fear of the COVID-19

Fear of the COVID-19 was measured by using the Fear of the COVID-19 Scale (FCV-19S) (Ahorsu et al., 2020). The FCV-19S contains seven items that measured FoC content with two domains: physical response of fear (four items) and fear thinking (three items). Items are measured on a 5-point Likert scale (from 1 = disagree to 5 = completely agree) where a higher mean score indicates higher FoC (Ahorsu et al., 2020). The previous study has validated the FCV-19S in different contents including Chinese content (Chi et al., 2021). The Cronbach’s alpha of the FCV-19S was 0.83.

### Depression

Depression was measured by using the Beck Depression Inventory-II (BDI-II) scale (Beck et al., 1996). The BDI-II contains 21 items that measured the level of depression in the recent 2 weeks; each item was rated on a 4-point scale (from 0 = symptom not present to 3 = symptom strongly present) with a higher mean score indicating a higher degree of depression (Beck et al., 1996). Wang et al. (2011) validated the BDI-II in Chinese content and the Cronbach’s alpha of the BDI-II scale was 0.80.

### Resilience

Resilience was assessed by using the Connor–Davidson Resilience Scale (CD-RISC) developed by Connor and Davidson (2003). The CD-RISC consists of 25 items rated on a 5-point Likert scale (from 0 = *not true at all* to 4 = *true nearly all the time*) with a higher mean score reflecting higher resilience (Connor and Davidson, 2003). The CD-RISC was validated in a well-used Chinese content by Yu and Zhang (2007) and the Cronbach’s alpha of the CD-RISC was 0.91.

### Social Support

Social support was measured by using the 6-item COVID-19 Version Perceived Social Support Questionnaire (F-SozU) developed by Sommerlad et al. (2020). This questionnaire is an adapted version of the F-SozU (Kliem et al., 2015) to measure perceived social support during the COVID-19 pandemic period. Each item was rated on a 5-point scale (from 1 = *not at all true* to 5 = *very true*) with a higher mean score reflecting the higher level of perceived social support. The Cronbach’s alpha of the F-SozU was 0.83.

### Statistical Analysis

The SPSS software version 26.0 was used for the data analysis. The demographic characteristics were analyzed by descriptive analyses. The Pearson correlation was calculated to test the bivariate correlations among FoC, resilience, social support, and depression. According to Hayes (2013), Model 4 and Model 1 in the PROCESS macro for SPSS (version 3.5.3) were used to test the mediating role of resilience as well as the moderating roles of resilience and social support. About 95% CIs of the indirect effects were calculated from 5,000 bootstrap resamples estimates in which mediating and moderating effects are significant at *p* < 0.05 when the CI does not include zero.

## Results

### Bivariate Correlations Among Study Variables

Descriptive statistics and correlations for all the variables are shown in Table 2. FoC was positively correlated with depression (*r* = 0.59, *p* < 0.001) and H1 was supported. Besides, FoC was negatively correlated with resilience (*r* = 0.37, *p* < 0.001) and social support (*r* = 0.24, *p* < 0.001). Both resilience (*r* = 0.52, *p* < 0.001) and social support (*r* = 0.42, *p* < 0.001) were negatively correlated with depression. Moreover, social support showed a significant positive correlation with resilience (*r* = 0.63, *p* < 0.001).

**TABLE 2:** Descriptive statistics and correlations between variables (*N* = 476).

	*M*	SD	1	2	3	4
1. Fear of COVID-19	15.94	5.99	1			
2. Resilience	62.97	11.77	−0.37***	1		
3. Social Support	3.51	0.61	−0.24***	0.63***	1	
4. Depression	10.11	5.30	0.59***	−0.52***	−0.42***	1

***p < 0.001.

### Role of Resilience

After controlling for sex, age, and education level, the mediating effect of resilience on the association between FoC and depression was analyzed. The results of the regression analysis are shown in Table 3.

**TABLE 3:** Regression analysis of relationship between fear of COVID-19 and depression with mediation analyses (*N* = 476).

		Coeff.	Standardized Coeff.	Boot Se	*p*	Bootstrap 95%CI		*R* ^2^	*F*
	
						Lower	Upper		
Resilience (Model l)								0.153	21.215***
	Constant	1.567		1.639	0.339	–1.655	4.788		
	Gender	–3.189	–0.129	1.053	0.003**	–5.257	–1.121		
	Age	1.171	0.059	1.178	0.321	–1.143	3.485		
	Education Level	–0.829	–0.042	1.191	0.486	–3.169	1.510		
	FoC	–0.705	–0.359	0.084	< 0.001***	–0.869	–0.540		
Depression (Model 2)								0.345	61.972***
	Constant	–0.664		0.649	0.307	–1.941	0.612		
	Gender	0.148	0.013	0.417	0.723	–0.672	0.967		
	Age	0.140	0.016	0.467	0.764	–0.777	1.057		
	Education Level	0.226	0.025	0.472	0.633	–0.701	1.153		
	FoC	0.516	0.583	0.033	< 0.001***	0.451	0.582		
Depression (Model 3)								0.453	77.792***
	Constant	–0.412		0.595	0.489	–1.581	0.757		
	Gender	–0.365	–0.033	0.385	0.344	–1.123	0.392		
	Age	0.328	0.037	0.427	0.443	–0.511	1.168		
	Education Level	0.092	0.010	0.432	0.831	–0.756	0.941		
	FoC	0.403	0.455	0.033	< 0.001***	0.339	0.467		
	Resilience	–0.161	–0.357	0.017	< 0.001***	–0.194	–0.128		

**p < 0.01, ***p < 0.001.

When sex, age, and education level were included in the regression model as three control variables, the results showed that FoC has a significant positive association with depression (β = 0.583, *p* < 0.001). Meanwhile, the negative association between FoC and resilience (β = 0.359, *p* < 0.001) as well as the negative association between resilience and depression (β = 0.357, *p* < 0.001) was also significant. Moreover, when resilience was used as a mediator, the positive association between FoC and depression (β = 0.455, *p* < 0.001) was also significant. The bootstrap results of the mediating effect indicated that the indirect effect of FoC on depression was significant (β = 0.113, *SE* = 0.017, 95% CI = 0.082, 0.148) and the mediating effect caused a variance of 21.89% of the total impact of the models (Table 4). As 95% CI in the path did not contain zero value, the results confirmed that H2 was supported.

**TABLE 4:** Resilience in the mediation effect analysis (*N* = 476).

	Effect	Boot SE	Bootstrap 95%CI		Effect%
	
			Lower	Upper	
Total effect	0.516	0.033	0.451	0.582	
Direct effect	0.403	0.033	0.339	0.467	78.10%
Indirect effect	0.113	0.017	0.082	0.148	21.89%

**FIGURE 2:**
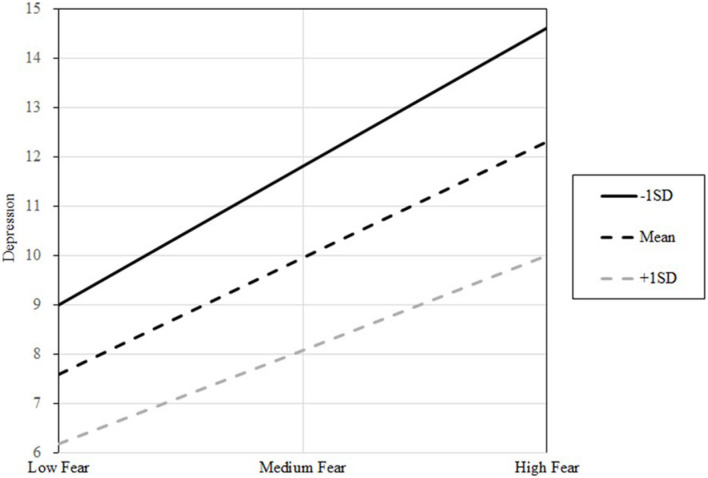
Results of the moderation model. Three lines are the visual representation of different moderation effects of FoC on depression when resilience scores were at its +1 SD, mean, and –1 SD. Fear refers to fear of COVID-19 (FoC).

To investigate the moderating effect of resilience and social support on the association between FoC and depression, the aforementioned covariates were controlled. Table 5 shows the interaction between FoC and depression from resilience emerged as a significant predictor [β = 0.006, *p* < 0.05, 95% CI 0.012; 0.001, *R*^2^ = 0.459, *F*(6,469) = 66.482, *p* < 0.001]. Additionally, changing *R^2^* = 0.007 statistically due to interactions, *F*(1,469) = 5.885, *p* < 0.05, which means that the moderation explained the 45.96% of the variance in the resilience of the model. Therefore, resilience has a positive significance on the relationship between FoC and depression. The results illustrated the interaction by depicting the regression line of the relationship between FoC and depression at high, medium, and low (+1 SD, mean, –1 SD) resilience scale scores. The graph showed that resilience played a role in narrowing the positive correlation between FoC and depression mentioned in the research literature. The highest levels of depression were found in the individuals who reported low levels of FoC and low resilience (Figure 2). Overall, the results of the moderation analysis indicated that the resilience of the individuals attenuated the association between FoC and depression in the COSs. Thus, H3 was supported.

**TABLE 5:** Results from a regression analysis examining the moderation of the effect of FoC on depression by resilience (*N* = 476).

Variables	Outcome: Depression				
	
	Coeff.	*SE*	*p*	LLCI	ULCI
Constant	–0.579	0.596	0.332	–1.750	0.592
FoC	0.394	0.033	< 0.001***	0.329	0.458
Resilience	–0.158	0.017	< 0.001***	–0.191	–0.126
FoC × Resilience	–0.006	0.003	0.016*	–0.012	–0.001
Gender	–0.391	0.383	0.308	–1.145	0.362
Age	0.329	0.425	0.439	–0.506	1.165
Education Level	0.104	0.429	0.809	–0.740	0.949
*R*^2^ = 0.4596					
*F*(6, 469) = 66.482, *P* < 0.001					

*p < 0.05 and ***p < 0.001.

### The Moderating Role of Social Support

After controlling for sex, age, and education level, the moderating effect of social support on the association between FoC and depression was analyzed. Contradictory to H4, the result showed that the moderating role of social support is insignificant. Being a predictor of FoC on depression, social support does not act as a moderator for the relationship [β = 0.002, *F*(6,469) = 58.8261, *p* = 0.956] (Table 6.). Thus, H4 was rejected.

**TABLE 6:** Results from a regression analysis examining the moderation of the effect of FoC on depression by social support (*N* = 476).

Variables	Outcome: Depression				
	
	Coeff.	*SE*	*p*	LLCI	ULCI
Constant	–0.132	0.115	0.249	–0.358	0.093
FoC	0.511	0.036	< 0.001***	0.440	0.582
Social Support	–0.301	0.036	< 0.001***	–0.372	–0.229
FoC × Social Support	–0.002	0.035	0.956	–0.069	0.066
Gender	–0.014	0.074	0.854	–0.159	0.131
Age	0.069	0.083	0.407	–0.094	0.231
Education Level	0.023	0.083	0.787	–0.141	0.186
*R*^2^ = 0.4294					
*F*(6,469) = 58.8261, *P* < 0.001					

***p < 0.001.

## Discussion and Conclusion

### Discussion

This study examined the mediating effect of resilience as well as the moderating effects of resilience and social support on the relationship between FoC and depression among the COSs studying online amid the COVID-19 pandemic period. The correlational analyses revealed that FoC had a positive relationship with depression, which was consistent with the previous studies that higher FoC is positively correlated with increased depression (Bakioğlu et al., 2020; Al Majali and Alghazo, 2021). The results indicated that FoC is an important trigger for depression among the COSs studying online amid the COVID-19 pandemic period. Marcus et al. (2014) argued that depression is a serious affliction that has a negative impact on the mental health of an individual. Thus, those COVID-19-related fears may increase the likelihood of depressive symptoms, which negatively influence the mental health of the COS.

This study also contributes to the theory by revealing the mechanism through which FoC influences the depression of the COSs studying online in China. Although previous research revealed that FoC is associated with depression, the underlying mechanism was not enough clear. Previous research indicated that mindfulness could help to counter the negative effects of FoC on depression, thus reducing depression (Belen, 2021). This study demonstrated the mediating role of resilience between FoC and depression. Previous research has shown that a higher level of FoC can restrain resilience, thus bombing the depression (Yıldırım et al., 2020), this study further examined that resilience is an effective mediator in the linkage between FoC and depression among the Chinese group. Resilience is mediated by assessing the path from FoC to depression. Weaker resilience is equipped when the individual experiences a higher-level fear mood. The situation of the COSs is different from those of Chinese students studying in the domestic universities in mainland China. They face the cultural differences and changing international circumstances after the COVID-19 outbreak that are complicated. At the same time, the institution of non-native languages and diverse curricula of outbound universities are stressful (Ching et al., 2017). Therefore, the COSs need the strong capability to adjust themselves to deal with fear, difficulty, and uncertainty. Resilience can meet all those requirements of the COSs ensuring that the COSs counter negative situations and enhance their mental health.

The findings of this study also showed that resilience moderated the relationship between FoC and depression, which is consistent with prior research showing that resilience has a negative and substantial impact on depression (Hjemdal et al., 2011). Individuals with weaker resilience are more likely to experience depressive symptoms. This finding further explains the resilience theory (Van Breda, 2001). Furthermore, those COSs studying online in China also need to endure time differences, network latency, and various emergencies while conducting their online learning. Under the special circumstances of the COVID-19 pandemic period, the COSs with higher resilience are more capable of dealing with social isolation, life stress (Cacioppo et al., 2011), and the threats of various crises (Kimhi et al., 2020). Thus, the COSs with stronger resilience may be better able to withstand the FoC and recover from its negative effects.

The present finding also contributes to the buffering effect in the buffering model of social support (Aneshensel and Stone, 1982). The moderating effect of social support on the association between FoC and depression was not sustained in this study. It was found that social support can be a preventive factor against negative psychological and emotional effects among Chinese students (Cheng, 1997; Chen, 2018). Previous studies also indicated that social support can be a preventive factor against negative psychological emotions among Chinese students (Cheng, 1997; Chen, 2018). The COSs are generally faced with high financial costs, high expectations from their families, heavy academic tasks, and not assimilated stereotypes of the Chinese group (Ruble and Zhang, 2013; Fang et al., 2020; Xi et al., 2020). In particular, the widespread prejudice against the China and Chinese citizens after the COVID-19 outbreak has put the COSs in a more complicated and hostile environment (Mittelmeier and Cockayne, 2020); thereby, social support is particularly imperative for them in this challenging situation. The reason why only a weak moderating effect was found could be that mere provision from the interpersonal resources is not sufficient to prevent the negative mental consequences under the predicament. Social support could only be beneficial if it is tailored to the most appropriate coping methods for the stressful circumstances (Sarason et al., 1990; Schreurs and de Ridder, 1997; André-Petersson et al., 2006). Therefore, future studies should focus more on the application environment of social support to provide the students with valuable helping in threatening those difficulties.

Fear of the COVID-19 has a tremendous mental health impact all over the world; therefore, related mental illness prevention is critical during this difficult time (Pakpour and Griffiths, 2020). While e-learning mode is employed worldwide during the COVID-19 pandemic period, this finding can provide empirical evidence in implementing psychological interventions to prevent depression among international students. As a result of their minority status, the mental health of the international students is often neglected by both their homeland and their studying region and they are more prone to depressive disorders (Chen et al., 2020). To help the international students to minimize their depression, FoC should be avoided that can be utilized as a preventive therapy. They could also be incorporated into the mental health training program of the college to assist the international students in building psychological resilience (Saltzman et al., 2020), hence minimizing depression.

Since FoC is a reaction to the worldwide spread of the COVID-19 (Daniel, 2020), this study can provide empirical data to verify the detrimental mental impact on the FOC as well as suggest novel models involving resilience, social support, and depression with FoC as a predictor. The findings of this study could contribute to understanding the buffering variables and the mental resources that are important to mitigate unfavorable consequences of mental health during the COVID-19 pandemic period. By suggesting that resilience may assist in minimizing depression, this study provided a novel research path for preventing mental health problems. Promoting internal resilience would be an effective strategy for reducing depression when faced with hardship (Brewin et al., 1989; Malhi et al., 2019).

### Implications

First, this study extends existing FoC and depression literature among the international student group by showing that FoC has a significant positive effect on the depression of the COS. A positive association between FoC and depression was found, which is consistent with previous studies showing that FoC can be a trigger to depression in the domestic contexts (Bakioğlu et al., 2020; Al Majali and Alghazo, 2021). Second, the findings indicated that FoC is associated with depression through both the mediation and moderation models with resilience. The current findings confirmed that resilience has significant implications in preventing the negative mental states under the COVID-19 context. The underlying mechanism of association between FoC and depression was explored. These findings also provided a more comprehensive grasp of mental health services and higher education institutions working with the international students to help determine the protocols and interventions to prevent mental illness in the students.

### Limitations and Future Directions

Some limitations that exist in this study need to be acknowledged. First, a previous study revealed that there are regional differences in FoC (Lo Coco et al., 2021). However, the different influences from the studying region of the COS were not applicable in this study. Second, the present sample of the COSs was predominantly composed of those studying in North America, the United Kingdom/European Union, or Hong Kong/Macau/Taiwan of China because of the commonly known study region preference of the COSs. Therefore, future studies should focus on the COSs from a specific region or obtain a more region-balanced sample to indicate the influence of FoC among the COSs studying in the different regions. Furthermore, this study did not collect the information on the current resident region in China of those COSs. Previous research has found that regional differences in various areas of China (e.g., comparing Wuhan with other cities in the low-risk infection areas) on the impact of the COVID-19 on mental health due to the epidemic history and risk of infection in these areas (Wen et al., 2020; Ge et al., 2021). Thus, a future study involving a regional difference in China could be conducted. Moreover, the study cannot directly emphasize the mechanisms of social support in preventing depression. Previous studies measured social support from different perspectives (Eagle et al., 2019; Ye et al., 2019) and perceived social support from different sources (Procidano and Heller, 1983). This study only measured the perceived social support during the COVID-19 pandemic period without detailed information on the support resources. Therefore, an additional measurement of social support that would be applied in the current results is not confirmed. On the other hand, the current study is only a cross-sectional design, which makes our findings that are not deep-going and comprehensive enough. Therefore, a longitudinal design that addresses the temporal association among those variables or an experimental design to control more confounding variables could be adopted in future studies. Fourth, due to the social distancing considering during the COVID-19 pandemic period, this study utilized an online self-reporting questionnaire; thus, interference from the external environment and potential response bias to the items were unavoidable. Therefore, multiple rating sources or measurements should be utilized to minimize the presence of bias during the data collection. Fifth, we use the Hayes approach (2013) to test the mediating and moderating roles of resilience and social support, which contains some potential limitations. Although this approach exhibits considerable statistical power, it is also more likely to make the error of type I than the other mediating and moderating methods (Preacher et al., 2011; Fang and Wen, 2018). Therefore, future research needs to take into account the errors caused by the statistical methods.

### Conclusion

This study demonstrated the mediating effect and the moderating effect of resilience on the relationship between FoC and depression among the COSs studying online in China during the COVID-19 pandemic period. However, the moderating effect of social support on the association between FoC and depression was not sustained in this study. The findings provided empirical evidence to confirm not only a positive relationship between FoC and depression but also suggest that it is mediated as well as moderated by resilience. Thus, providing more research evidence to attention in resilience training from institutions in higher education because of their significant role in the mental health of the international students.

## Data Availability Statement

The datasets generated for this study are available on request to the corresponding author.

## Ethics Statement

The studies involving human participants were reviewed and approved by the Research Ethics Sub-Committee of the College of Liberal Arts and Social Sciences in the City University of Hong Kong. The patients/participants provided their written informed consent to participate in this study.

## Author Contributions

YC designed the research and completed the manuscript writing. YC, YL, and ZL collected and analyzed the data. YZ and TZ reviewed and edited the manuscript. All authors contributed to the article and approved the submitted version.

## Conflict of Interest

The authors declare that the research was conducted in the absence of any commercial or financial relationships that could be construed as a potential conflict of interest.

## Publisher’s Note

All claims expressed in this article are solely those of the authors and do not necessarily represent those of their affiliated organizations, or those of the publisher, the editors and the reviewers. Any product that may be evaluated in this article, or claim that may be made by its manufacturer, is not guaranteed or endorsed by the publisher.
